# Granular Cell Tumor: A Rare Entity Occurring at an Uncommon Site

**DOI:** 10.7759/cureus.90699

**Published:** 2025-08-21

**Authors:** Yousef Almutairi, Humoud Al-Sabah, Alsadat Mosbeh

**Affiliations:** 1 Dermatology, Al Jahra Hospital, Al Jahra, KWT; 2 Dermatopathology, As'ad Al-Hamad Dermatology Center, Kuwait City, KWT; 3 Dermatology/Dermatopathology, Faculty of Medicine, Al-Azhar University, Cairo, EGY

**Keywords:** abrikossoff tumor, granular cell tumor, schwann cell origin, vulva, vulvar neoplasm

## Abstract

Granular cell tumor (GCT), also known as Abrikossoff tumor, is an uncommon clinical entity derived from Schwann cells. Most are found in the oral cavity, especially the tongue. However, in this case, a rare clinical presentation occurred on the vulva of a young female patient, who presented with a progressively enlarged solitary nodule on her left labia majora. Histological examination showed a nonencapsulated tumor in the dermis, composed of large cells with abundant granular cytoplasm. Immunohistochemical staining revealed strong expression of S100 by tumor cells. Based on histopathological results, the vulvar tumor has been completely resected under local anesthesia with clear surgical margins of 0.5 cm. The patient has shown no tumor recurrence after six months following the surgery. In short, we think that GCT should be considered as one of the differential diagnosis of a solitary genital tumor at young ages.

## Introduction

Granular cell tumor (GCT) is a rare neoplasm first characterized by Abrikossoff in 1926 [1]. The origin of the tumor remains uncertain, with multiple theories proposed. The initial designation of myoblastoma was based on the apparent resemblance of granular cells to skeletal muscle cells in paraffin sections [2]. However, subsequent electron microscopy and immunohistochemical analyses ruled out this possibility, leading to the favored hypothesis that the tumor is related to Schwannian neoplasms [3]. GCT accounts for approximately 0.5% of all soft tissue tumors [4]. This type of tumor can be seen at any age, although most commonly in adults. It is important to note that children may be affected [5]. Only 1%-3% of GCT is malignant with local infiltration, recurrences, and distant metastases [6]. It typically presents as a painless soft nodule arising on the head and neck, preferably the tongue. Vulvar involvement is rare, being only 5%-16% of the cases [7]. The clinical diagnosis of vulvar GCT is quite difficult, as its appearance often mimics that of more common genital lesions.

Here, we report a case of GCT on the left labia majora in a young female patient, which was successfully treated with surgical excision. Although rare, GCT should be considered in the differential diagnosis of a solid vulvar tumor.

This article was previously presented as a meeting abstract at the 28th Joint Meeting of the International Society of Dermatopathology on March 6, 2025.

## Case presentation

**Figure 1:**
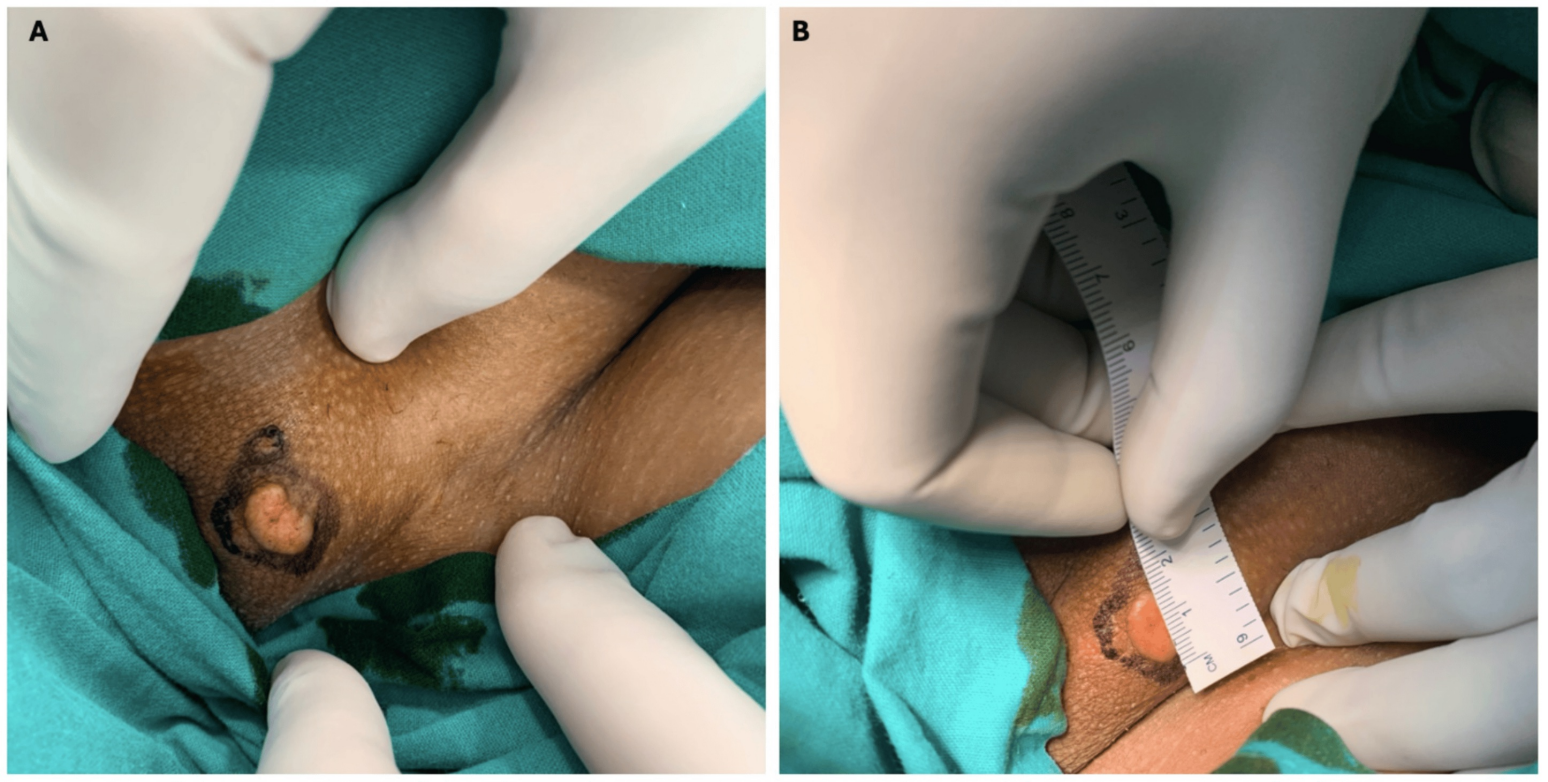
(A,B) A granular cell tumor located on the left labia majora in a 28-year-old woman

A 28-year-old woman, known case of glucose-6-phosphate dehydrogenase deficiency, presented with a nine-month history of a gradually enlarging, mildly pruritic nodule on the genitalia. The lesion was initially small, skin-colored, and asymptomatic until recently, when the patient noticed a sudden increase in its size. There was no history of trauma, discharge, or bleeding from the lesion. There was a history of a genital wart in her husband, and there was no significant family history. The lesion had previously been managed as condyloma acuminatum, but without significant improvement. On physical examination, a solitary, hypopigmented, nontender, and firm nodule with a verrucous surface was located on the left labia majora, measuring approximately 1.5 × 1.0 cm in size (Figure 1). No ulceration, crusting, or other signs of secondary infection were noted. A full body physical examination revealed no other lesions elsewhere on the body and no palpable lymph nodes.

**Figure 2:**
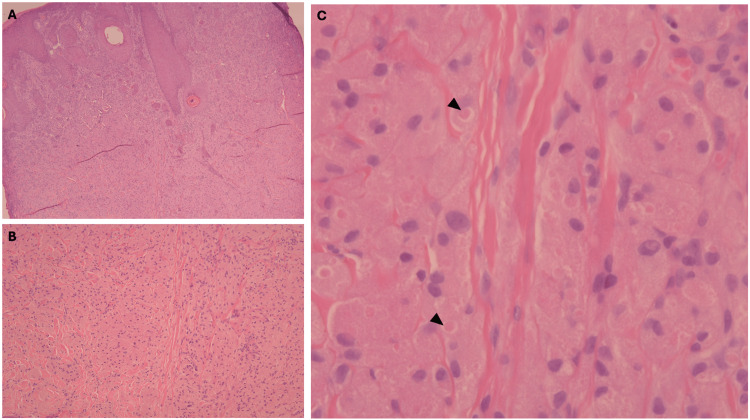
Histopathological examination of the skin biopsy (hematoxylin and eosin). (A,B) A pseudoepitheliomatous hyperplasia with a nonencapsulated tumor in the dermis. (C) Tumor cells with copious granular and eosinophilic cytoplasm with pustulo-ovoid bodies of Milian (arrows)

**Figure 3:**
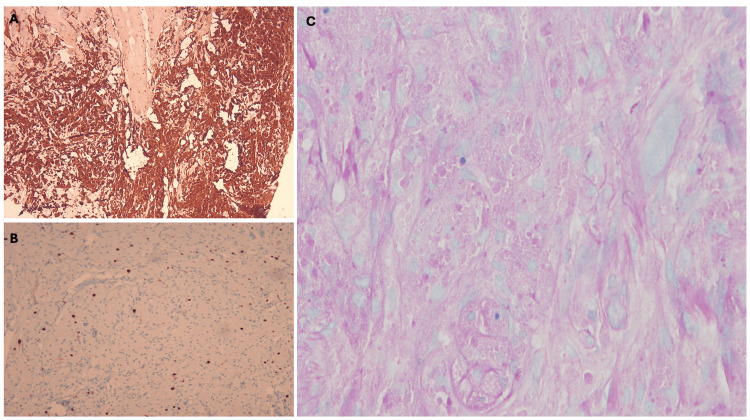
Immunohistochemistry slides. (A) Cells stained strongly with S100. (B) Very low Ki-67 labeling index (<5%). (C) Cytoplasmic granules were positive for PAS PAS: periodic acid-Schiff

The patient was advised to undergo an incisional biopsy to rule out serious tumors such as squamous cell carcinoma. Histopathological examination of the biopsy showed marked acanthotic epidermis overlying a nonencapsulated tumor in the dermis and subcutaneous tissue. The tumor is composed of large polygonal cells with a small hyperchromatic nucleus and abundant eosinophilic granular cytoplasm. In some places, some granules had coalesced into larger structures known as pustulo-ovoid bodies of Milian, which were surrounded by a clear halo (Figure 2). Immunohistochemical staining revealed a strong cytoplasmic expression of S100 by the tumor cells, confirming the diagnosis of GCT. The cytoplasmic granules were positive for periodic acid-Schiff (PAS) staining, and the Ki-67 proliferation index was low (<5%) (Figure 3).

**Figure 4:**
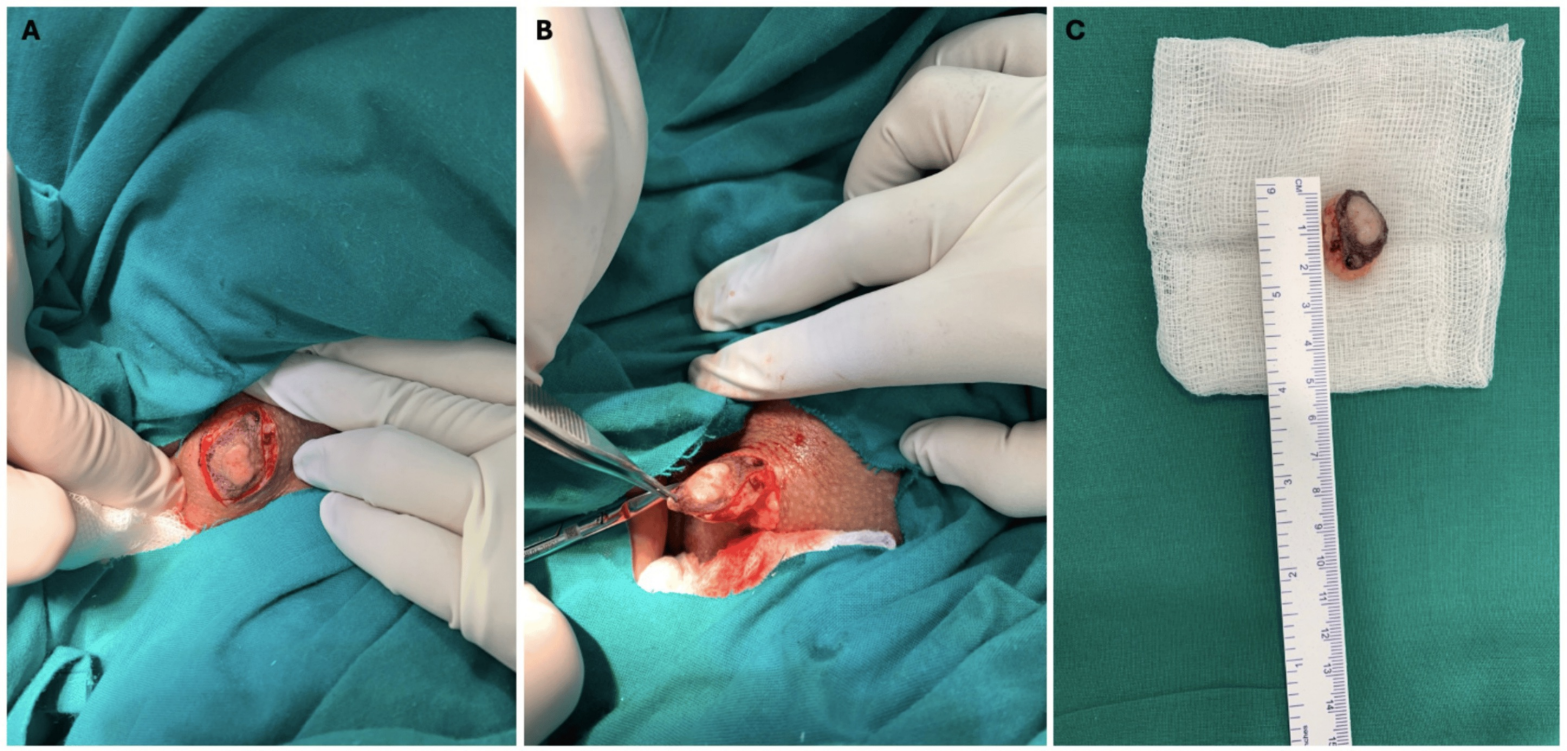
(A,B) The granular cell tumor of the left labia majora during complete surgical excision with clear margins of 0.5 cm. (C) Tumor after complete excision

Based on the biopsy results, the patient underwent complete excision of the vulvar tumor under local anesthesia with clear margins of 0.5 cm (Figure 4). At the six-month follow-up, the patient showed no evidence of local recurrence.

## Discussion

GCT is a rare and predominantly benign tumor that can arise in any part of the body. As of 1999, approximately 400 cases have been reported in the literature [8]. Various cell types have been implicated in the histogenesis of granular cell tumor (GCT); however, most histochemical and ultrastructural evidence supports a Schwann cell origin [3]. This tumor has been observed in patients of all ages, including children. In adults, the average age of distribution is between the fourth and sixth decades [9]. While common sites are the head and neck, vulvar involvement is rare and has been reported in around 5%-16% of cases [7]. Although GCT usually behaves benignly, there is a very low risk of malignant transformation, reported in approximately 1%-3% of cases [6]. When this occurs, there may be a chance for metastasis to lymph nodes or other distant tissues [10].

GCT usually presents as an asymptomatic and slow-growing, small submucous, or subcutaneous nodule [11]. Overlying skin may be depigmented, thickened, or even ulcerated [12]. Hypertrichosis of a GCT has been reported [13]. Clinical diagnosis may be challenging due to the tumor’s resemblance to various benign and malignant lesions. The tumor may be mistaken for benign fibroadipose lesions, adnexal neoplasms, bartholin duct cysts, epidermal inclusion cysts, and even squamous cell carcinoma.

Histopathologically, GCT is composed of large polygonal cells with abundant eosinophilic and granular cytoplasm. The granular appearance results from the accumulation of secondary lysosomes within the cytoplasm. These granules are PAS-positive and diastase-resistant. Immunohistochemically, GCTs are positive for S100, neuron-specific enolase, and vimentin, while negative for cytokeratin, melanoma antigen (Melan-A), desmin, CD31, and CD34 markers. This immunohistochemical profile is consistent with a Schwann cell origin [3,14]. The overlying epithelium often exhibits prominent pseudoepitheliomatous hyperplasia, which can be mistaken for squamous cell carcinoma if only a superficial biopsy is obtained for histopathological evaluation [15].

Histopathological features associated with malignant transformation have been investigated in several studies. Fanburg-Smith et al. proposed a classification system for GCTs based on six criteria [16]: 1) the presence of necrosis, 2) the presence of spindle-shaped tumor cells, 3) vesicular nuclei with prominent nucleoli, 4) increased mitotic activity (greater than or equal to two mitoses per 10 high-power fields), 5) a high nuclear-to-cytoplasmic ratio, and 6) marked cellular pleomorphism. According to this classification, tumors lacking all six features are considered benign; those exhibiting three or more criteria are classified as malignant, while tumors meeting one or two criteria are designated as atypical. In challenging cases, Ki-67 labeling index can be of support, showing a significant increase in tumors with malignant behavior compared to benign ones [17]. No evidences of malignancy were detected in our case.

Treatment of GCT consists of complete surgical excision with disease-free margins, followed by close clinical follow-up [18]. The risk of local recurrence is approximately 2%-8% when surgical margins are negative; however, this risk may increase to up to 20% in cases with positive margins [19].

## Conclusions

GCT is a rare neoplasm, and its occurrence in the vulvar region is particularly uncommon. While GCTs are mostly benign, malignant transformation can occur, albeit at a low rate. It typically presents as an asymptomatic papule or nodule. A thorough clinical evaluation of the entire body is recommended to rule out multifocal lesions. Immunohistochemistry is a crucial tool for establishing a definitive diagnosis. The primary treatment approach is complete surgical excision with clear margins. The prognosis is generally favorable, with a low rate of recurrence. GCT should be considered in the differential diagnosis of both benign and malignant vulvar tumors.
